# Fatigue Behavior of Glass Fiber-Reinforced Polymer Bars after Elevated Temperatures Exposure

**DOI:** 10.3390/ma11061028

**Published:** 2018-06-16

**Authors:** Guanghui Li, Jun Zhao, Zike Wang

**Affiliations:** 1 School of Civil Engineering, Zhengzhou University, Zhengzhou 450001, China; lgh0524@126.com; 2 School of Mechanics and Engineering Science, Zhengzhou University, Zhengzhou 450001, China; zkwang@zzu.edu.cn

**Keywords:** glass fiber-reinforced polymer (GFRP) bars, elevated temperature, cyclic load, tensile strength, elastic modulus, fatigue behavior

## Abstract

Fiber-reinforced polymer (FRP) bars have been widely applied in civil engineering. This paper presents the results of an experimental study to investigate the tensile fatigue mechanical properties of glass fiber-reinforced polymer (GFRP) bars after elevated temperatures exposure. For this purpose, a total of 105 GFRP bars were conducted for testing. The specimens were exposed to heating regimes of 100, 150, 200, 250, 300 and 350 °C for a period of 0, 1 or 2 h. The GFRP bars were tested with different times of cyclic load after elevated temperatures exposure. The results show that the tensile strength and elastic modulus of GFRP bars decrease with the increase of elevated temperature and holding time, and the tensile strength of GFRP bars decreases obviously by 19.5% when the temperature reaches 250 °C. Within the test temperature range, the tensile strength of GFRP bars decreases at most by 28.0%. The cyclic load accelerates the degradation of GFRP bars after elevated temperature exposure. The coupling of elevated temperature and holding time enhance the degradation effect of cyclic load on GFRP bars. The tensile strength of GFRP bars after elevated temperatures exposure at 350 °C under cyclic load is reduced by 50.5% compared with that at room temperature and by 36.3% compared with that after exposing at 350 °C without cyclic load. In addition, the elastic modulus of GFRP bars after elevated temperatures exposure at 350 °C under cyclic load is reduced by 17.6% compared with that at room temperature and by 6.0% compared with that after exposing at 350 °C without cyclic load.

## 1. Introduction

As a new type of structural material in civil engineering, fiber-reinforced polymer (FRP) has become an important supplement to traditional materials in civil engineering due to its excellent mechanical, physical and chemical properties [1,2,3]. At present, FRP composite materials in civil engineering mainly include four categories based on the form of application: FRP strengthened structures, FRP ribs and cables to replace steel bars and steel cables, FRP combined with traditional materials and full FRP structures [4,5,6,7,8]. Furthermore, as a form of FRP composite materials, FRP bars are attractive for wide use in civil engineering structures especially where steel bars and/or pre-stressed steel bars are not suitable due to highly corrosive environments or where an electromagnetic transparency of structure are required [9,10,11,12]. In the past two decades, a large number of studies have focused on FRP bars in reinforced concrete applications [13,14,15].

At room temperature, the mechanical properties of FRP reinforced concrete structures can satisfy both the ultimate limit state and serviceability limit state requirements [16,17]. Considering that the FRP bars are non-metallic composite materials composed of fiber and resin matrix, the fibers (as inorganic compounds) are very stable and mostly insensitive to temperature variations. When the working temperature was lower than the glass-transition temperature of the resin, glass fiber-reinforced polymer (GFRP) materials can still exhibit excellent performance compared to other metallic materials [18]. However, the epoxy polymer matrix will be vitrified and carbonized when subjected to elevated temperature exposure, which leads to the degeneration and loss of its cohesive action gradually [19,20]. Moreover, since the properties of the continuous fiber material will be reduced after the elevated temperature exposure, the mechanical properties of the FRP bars decrease after exposed to fire or elevated temperatures, which finally affects the mechanical properties of the FRP reinforced concrete structures subjected to elevated temperatures.

With the large number of applications of FRP bars in engineering, many researchers have made a series of investigations on the physical and mechanical properties of FRP bars at elevated temperature and after elevated temperature. The study of Robert and Benmokrane [20] showed that the ultimate tensile strength and elastic modulus of GFRP both decreased with the increasing exposure temperature, and once the exposure temperature exceeded the glass-transition temperature (120 °C) of the polymer matrix, the mechanical properties, especially the stiffness and the strength of GFRP bars decreased considerably. When the exposure temperature exceeded 300 °C, the ultimate tensile strength of GFRP bars was about 46% lower than that at room temperature (25 °C). The test results of Devon et al. [21] indicated that the residual tensile strength of GFRP bars at 400 °C was 83% of that at room temperature, which is highly different from Reference [20] with the decrease of 46% of the tensile strength of GFRP bars at 300 °C. Similarly, Wang et al. [22] found that the ultimate tensile strength of GFRP bars was reduced by a maximum of 6% at less than 80 °C, and by 22% at 80–120 °C. Further, the ultimate tensile strength of GFRP bars decreased sharply at 400 °C, and reduced by as high as 67% at 500 °C. Alsayed et al. [23] investigated the influence of heating time (1, 2 or 3 h) on tensile properties of GFRP bars after different elevated temperatures exposure (100 °C, 200 °C, 300 °C). The result showed that the reduction rate of the tensile elastic modulus of GFRP bars was within 5%, while that of the tensile strength of GFRP bars was in the 9.7–41.9% range. Meanwhile, the reduction of strength was linearly related to the elevated temperature and heating time. Furthermore, Wang et al. [24] compared the tensile properties of CFRP and GFRP tendons under elevated temperatures. The experimental results showed that the elastic modulus and tensile strength of CFRP tendons decreased by 14% and 42% respectively at 200 °C, and the tensile strength of CFRP tendons at 400 °C was reduced by 70% compared to that of untreated CFRP tendons at the room temperature. Finally, the bearing capacity of CFRP tendons was almost lost at more than 500 °C. However, the elastic modulus of GFRP bars remained 90% of its initial value (i.e., at room temperature condition) at 400 °C, while decreased sharply at more than 400 °C. Similarly, Hamad et al. [25] investigated the mechanical properties of FRP bars with different types of fibers at elevated temperatures. The results showed that the FRP bars suffered significant reductions in their mechanical properties upon exposure to high temperatures of up to 450 °C at which the GFRP and BFRP was melted and lost their total tensile strength capacity. At a critical temperature of 325 °C, the tensile strength and elastic modulus of FRP bars lost as high as 55% and 30% respectively. In addition, the tensile tests of BFRP and GFRP bars with different diameters and elevated temperatures were carried out by Li et al. [26]. The results showed that the degradation of both FRP bars was not obvious at lower than 120 °C. At 120–270 °C, the tensile strength of BFRP bars tended to decrease less than that of GFRP bars. However, the elastic modulus of both FRP tendons was less affected by the high temperature within 270 °C. When the temperature reached 350 °C, the tensile properties of both FRP bars were drastically reduced and the bearing capacity was almost lost. Therefore, due to different factors such as production process and level, resin types, fiber types, heating curve, and period of heating, the properties of FRP bars showed different degrees of deterioration after elevated temperatures [21,22,25,26]. 

At present, many researchers have carried out experimental research on fatigue properties of FRP bars and concrete members with FRP strengthened [27,28,29,30,31,32], which mainly focused on the fatigue properties of FRP bars/concrete members with FRP strengthened at room temperature. Refai [33] found that there was a significant difference in the fatigue life for FRP bars with different fiber types at the same stress level. The fatigue life of CFRP bars was the longest. The fatigue life of FRP bars decreased by orders of magnitude with the increase of stress range. Demers [34] also obtained the similar research results. Noel et al. [35] compared the fatigue performances of GFRP bars in air and embedded in concrete. The experimental findings showed that the survived fatigue lives of GFRP bars in air were approximately a full order of magnitude longer than those of similar bars in concrete beams. Adimi et al. [36] reported that the logarithm of fatigue life decreased linearly with the increasing loading frequency. Nakada et al. [37] studied the long-term fatigue life of five kinds of FRP laminates combined with matrix resin, fiber and fabric for marine use under temperature and water environments. The results showed that the flexural fatigue strength of CFRP laminates with epoxy resin and GFRP laminates with vinyl ester resin both decreased with chemical reaction of matrix due to the process of water absorption and re-drying. Thus, it can be seen that the types of fiber, stress range, fatigue test method, test environment conditions and loading frequency are the important influence factors on the fatigue properties of FRP bars. 

However, to the authors’ best knowledge, the previous findings mainly focused on the mechanical properties of FRP bars under short-term monotonic loading after elevated temperature exposure and the fatigue properties of FRP bars/concrete members with FRP strengthened at room temperature. There was little research on the fatigue mechanical properties of FRP bars after exposure to elevated temperatures. To fill this research gap, this paper was to investigate the effect of elevated temperature with a range from 25 °C to 350 °C on the fatigue properties of GFRP bars. The fatigue behaviors of GFRP bars (including tensile strength, elastic modulus, and failure modes and so on) were considered to be investigated after elevated temperatures exposure. The outcome of this investigation can be used by future researchers and engineers for evaluating fire resistance of concrete structural members reinforced with GFRP bars when subjected to elevated temperatures.

## 2. Experimental Section 

### 2.1. Materials

The bars adopted in this study were 12 mm diameter (with a cross-sectional area of 113.04 mm^2^). The tensile properties of GFRP bars used were given in Table 1.

### 2.2. Test Equipment

A high-temperature furnace was used as the heating equipment in this study. The dimension of high-temperature furnace (L × W × H) is 1800 mm × 250 mm × 400 mm, with the heating rate of 10 °C/min and the maximum operating temperature of 1000 °C. Using control instruments, the high-temperature furnace can automatically achieve heating, insulation and shutdown. 

A 100 kN electro-hydraulic servo dynamic and static universal testing machine with a loading frequency of 1–10 Hz was used as the fatigue tensile test equipment. 

### 2.3. Experimental Design

In fact, for GFRP bars applied in concrete structures, the coupling of sustained load and elevated temperature on GFRP bars should be considered. Therefore, it is more realistic to examine temperature elevation of bars being under stress and then perform fatigue load at room temperature. However, due to the limitation of the test setup, it is noted that only the effect of elevated temperature was investigated on the fatigue property of GFRP bars in this study. This will be as the basis of the coupling of elevated temperature and sustained stress on fatigue property of GFRP bars. 

To obtain statistically relevant test results, 8 specimens were prepared. They were tested to failure from each set of FRP bars for different temperature exposure levels ranging from room temperature (25 °C) to 350 °C and for a holding time of 0, 1 or 2 h, of which 3 specimens were subjected to static load and 5 specimens were subjected to fatigue load. Hence a total of 105 specimens were prepared and tested in this study. The designations of tested specimens are presented in Table 2. 

### 2.4. Preparation of Specimens

The GFRP bars were designed and manufactured according to the American specification ACI 440.3R-04 [38]. The tensile samples were cut to 1200 mm in length with anchor length of 300 mm and the testing length of 600 mm. Firstly, according to the predetermined elevated temperature and holding time, the test samples were heated up with heating rate of 10 °C/min, opening the door of high-temperature furnace after the completion of heating. Secondly, the tensile samples were prepared after cooling to room temperature. Considering the low gripping resistance on the surface of GFRP bars, the design of an appropriate anchorage system was the key issue. To make sure that the failure of the FRP bars occurred in the middle part of GFRP specimens rather than at the anchorages in the test, the circular steel tubes grouted with expansive cement were used to mount the both ends of GFRP bars [39]. The length of the anchorage and the size of the galvanized steel tube were the key parameters in the design of the anchorage system. The nominal dimensions of the galvanized steel tubes were 34 mm in outer diameter, 3 mm in thickness and 300 mm in length. To prevent torque and bending during loading, the FRP specimens were aligned vertically and centrally in the anchorage tubes [24].

### 2.5. Experiment Method

According to the American specification ACI 440.3R-04 [38], the effect of cyclic load on the tensile strength and elastic modulus of GFRP bars was investigated, and the stress-strain curve and elastic modulus of GFRP bars were measured using the extensometer. It was noted that to avoid the damage of extensometer during the tensile test, only the elastic stage of the stress-strain curves for all GFRP bars were only recorded in this study. Five fatigue cycles (4000, 6000, 8000, 10,000 and 14,000) were adopted to investigate the effect of cyclic load on the tensile strength and elastic modulus of GFRP bars. Once the required fatigue cycles were reached, the fatigue loading stop and then the tensile test of GFRP bars was conducted immediately. The test results were compared with the initial tensile result of GFRP bars without the cyclic load. The maximum stress of fatigue test was 243 MPa (i.e., 30% of ultimate tensile strength), the minimum stress of fatigue test was 109 MPa (i.e., 13% of ultimate tensile strength), the stress range was 134 MPa (i.e., 17% of ultimate tensile strength), the cyclic stress rate was σ_min_/σ_max_ = 0.45, and the loading frequency was 3 Hz. Noted that the fatigue life of unexposed GFRP bars under above test parameters at room temperature was approximately 21,000 times. This lower fatigue life (much less than the required 2 million cycles) was much close to the higher stress range adopted in this study [33].

## 3. Test Features

### 3.1. The Appearances of GFRP Bars after Elevated Temperatures Exposure

**Figure 1:**
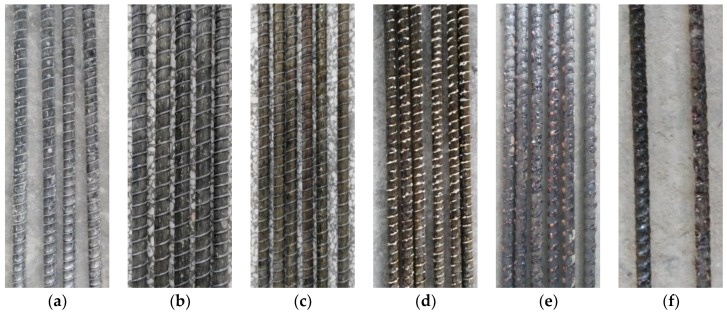
Appearances of GFRP bars after elevated temperatures exposure: (**a**) at 100 °C and holding time of 2 h; (**b**) at 150 °C and holding time of 0 h; (**c**) at 200 °C and holding time of 2 h; (**d**) at 250 °C and holding time of 0 h; (**e**) at 300 °C and holding time of 1 h; (**f**) at 350 °C and holding time of 0 h.

Figure 1 shows the apparent characteristics of GFRP bars after different elevated temperatures exposure and holding times. At 100 °C and the holding time of 0 h and 1 h, there was no obvious change on the surface of GFRP bars compared with the specimens at room temperature. When the holding time was 2 h, as shown in Figure 1a, the surface of the GFRP bars was whitened and the resin was melted. Meanwhile, the ribs on the surface of the GFRP bars were separated from the substrate or not firmly bonded. At 150 °C and the holding time of 0 h, as shown in Figure 1b, the surface of GFRP bars tended to be slightly yellow and the resin flowed downward. At 200 °C and the holding time of 2 h, the surface of specimen was golden yellow, and the phenomenon of the flow of resin became more serious (Figure 1c). At 250 °C and the holding time of 0 h, the surface of the test piece gradually changed from yellow to black and the resin was vitrified, and the ribs of GFRP bars also began to melt (Figure 1d). At 300 °C and the holding time of 1 h, the surface of the test piece was black and red, and the carbonization of resin was serious (Figure 1e). Finally, as shown in Figure 1f, at 350 °C and the holding time of 0 h, the surface of the test piece was burnt to black, and the destruction of the resin matrix was grievous. These results proved that the degradation degree of the surface part of GFRP bars was highly sensitive to the exposure temperature. Similar phenomena for GFRP bars after elevated temperatures were also found in previous literatures [23,25].

### 3.2. Fatigue Failure Characteristics

As found in the tensile fatigue test of GFRP bars, at the beginning, the sound of subtle fiber breaks can be heard occasionally during fatigue loading. Before the fatigue failure, the sound of the fiber fracture broke out, and in a very short time, the GFRP bars were damaged. The whole fatigue failure process of GFRP bars can be summarized as four stages [40]: (1) matrix cracking, (2) crack coupling interfacial debonding, (3) fiber breaking, (4) fracture. At the initial stage of cyclic load, micro-cracks began to appear in the matrix resin. With the increase of the number of cyclic load, the micro-cracks within the substrate of the bars gradually expanded and were close to the surface of GFRP bars due to the fatigue damage. Then the stress concentration zone on the tip end of the matrix cracks were formed on the surface of GFRP bars. The increase of the cycle times aggravated the stress concentration and stress redistribution, which led to the fracture of a small amount of fiber bearing load. Meanwhile, the load was redistributed in the rest of the fiber, which made the stress of the fiber increase obviously. Finally, the deterioration continued to develop until the fracture of GFRP bars broke down. 

**Figure 2:**
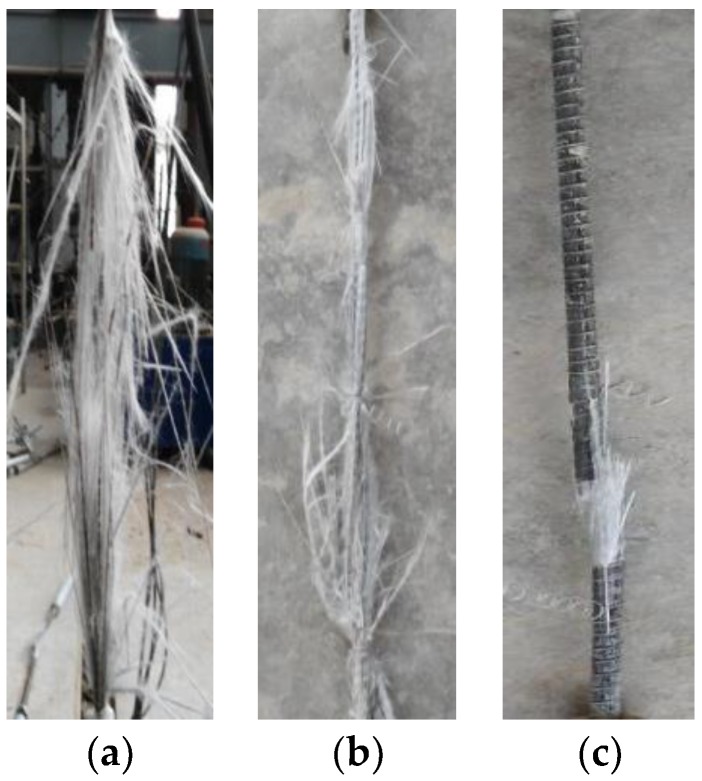
Failure modes of GFRP bars under cyclic load: (**a**) form of blasting damage; (**b**) form of splitting damage; and (**c**) form of fracture failure.

The failure modes after elevated temperature exposure were similar to the tensile failure characteristics at room temperature [26]. There were three types of failure modes of the tensile fatigue test of GFRP bars after elevated temperature exposure, namely blasting, splitting and fracture. All these three kinds of tensile fatigue failure modes of GFRP bars are presented in Figure 2. As found during the fatigue test, the stress uniformity of fiber interlayer played an important role on the fatigue failure mode of GFRP bars in the test length range. When the glass fibers of GFRP bars broke down almost simultaneously, the specimen took the form of blasting damage (Figure 2a). When the uneven force distribution occurred among the fibers of GFRP bars, the fiber broke at different sections, then the specimen damaged in the form of splitting (Figure 2b). The fracture failure of GFRP bars was due to the severe stress concentration at a section caused by the fatigue load. It was noted that for the fracture failure, there was no obvious damage at other sections of the GFRP bars (Figure 2c).

## 4. Results and Discussion

### 4.1. Tensile Properties of GFRP Bars after Elevated Temperature Exposure

#### 4.1.1. Effect of Elevated Temperature and Holding Time on Tensile Strength of GFRP Bars

**Figure 3:**
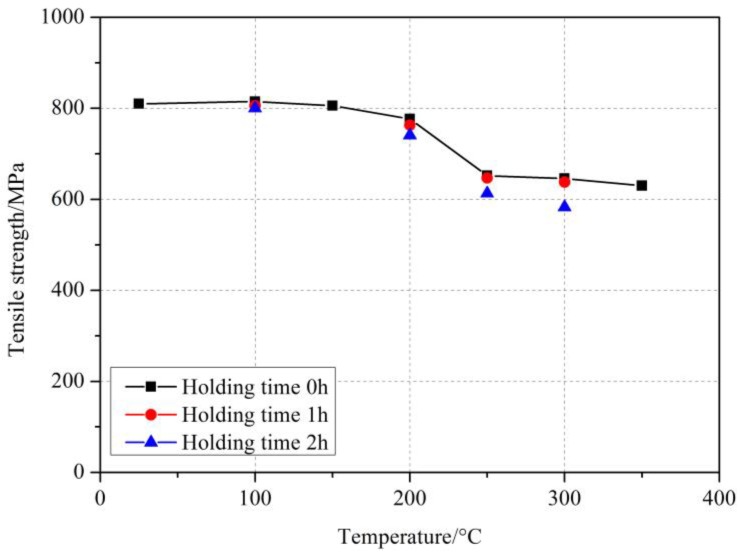
Tensile strength of GFRP bars after elevated temperature exposure.

Figure 3 shows the tensile strength of GFRP bars after different elevated temperatures and holding time. It can be seen from Figure 3 that with the increase of elevated temperature, the tensile strength of GFRP bars decreased gradually. At less than 150 °C, the tensile strength of GFRP bars was not significant reduction from that of GFRP at room temperature. In this condition, because the temperature was not too high, and GFRP bars were exposed to elevated temperature for a short period of time, the polymeric matrix on the surface of the GFRP bars was not vitrified. Besides, the elevated temperature had little effect on the tensile strength of glass fiber. Therefore, the deterioration of GFRP bars was not obvious. When the temperature reached 200 °C, the tensile strength of GFRP bars began to decrease, which decreased by 4.1% compared with that at room temperature. At 250 °C, the tensile strength of the GFRP bars decreased significantly, which was reduced by 19.5%. With the temperature increasing to 300 °C and 350 °C, the tensile strength of GFRP bars decreased by 20.2% and 22.2%, respectively. Thus, for the GFRP bars in this study, 250 °C can be considered as the critical temperature exceed which the tensile strength begins to drop significantly [20]. This result was basically consistent with the appearance results of GFRP bars, as Figure 1 also showed that the surface appearance of GFRP bars started to suffer the serious damage at more than 250 °C.

In addition, as can be seen from Figure 3, the holding time also had a certain deterioration effect on the tensile strength of GFRP bars subjected to elevated temperatures. For a certain temperature, the degradation of GFRP bars became more severe with the increase of holding time, and the tensile strength of GFRP bars decreased more obviously. However, at different elevated temperatures, the degree of deterioration of GFRP bars was varied with the holding time. The higher the elevated temperature was, the more obvious the deterioration of holding time on the tensile strength of GFRP bars was. At 100 °C, the tensile strength of GFRP bars decreased by 0.5% and 1.2% at holding time of 1 h and 2 h, respectively. While at 200 °C, the tensile strength of GFRP bars decreased by 5.8% and 8.5% at holding time of 1 h and 2 h, respectively. Further, at 300 °C, the tensile strength of GFRP bars decreased by 21.2% and 28.0% at holding time of 1 h and 2 h, respectively. These results showed that the tensile strength of GFRP bars was not significantly reduced at 100 °C with the increase of holding time, the holding time could be neglected at the relatively low 100 °C. When the temperature reached 200 °C, the deterioration effect of holding time on the tensile properties of GFRP bars increased gradually.

#### 4.1.2. Effect of Elevated Temperature and Holding Time on Elastic Modulus of GFRP Bars

**Figure 4:**
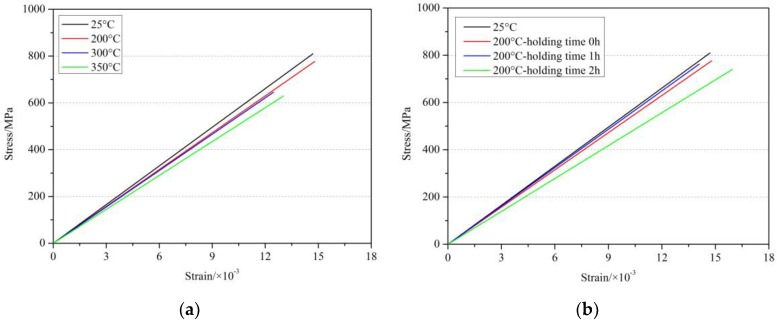
Stress-strain curve of GFRP bars after elevated temperatures exposure: (**a**) at holding time of 0 h; (**b**) at 200 °C.

**Figure 5:**
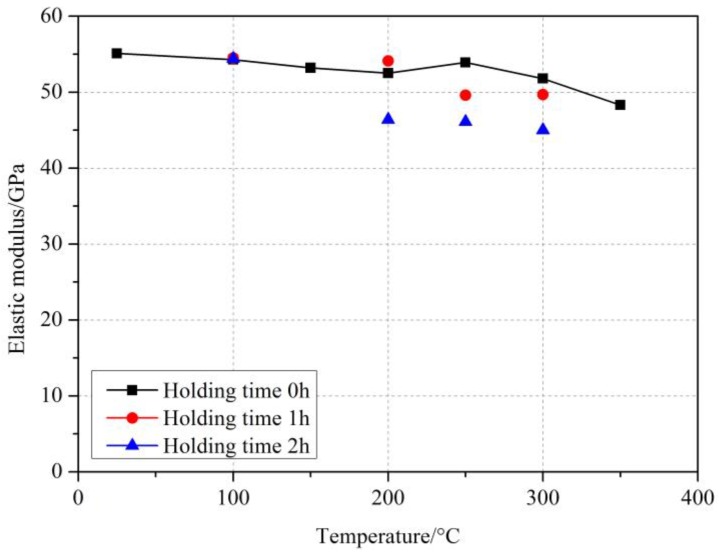
Elastic modulus of GFRP bars after elevated temperatures exposure.

Figure 4 summarizes the stress-strain curves of GFRP bars at room temperature and after different elevated temperatures exposure. It can be seen from Figure 4 that all the stress-strain curves of GFRP bars also tended to be typical linear even after elevated temperatures exposure. Similar results were also found in [24,25,26]. Further, Figure 5 showed the elastic modulus values of GFRP bars after different elevated temperatures and holding time. It can be seen from Figure 4 and Figure 5 that the elevated temperatures and holding time had deterioration influences on the elastic modulus of GFRP bars, and the degree of deterioration was related to the elevated temperature and holding time. At the temperature within 300 °C with the holding time of 0 h, the effect of elevated temperature on the elastic modulus of GFRP bars was slight and the change rate of elastic modulus was less than 6.0%. Until the temperature reached 350 °C, the elastic modulus decreased by 12.3%. In addition, holding time had a greater effect on the elastic modulus. At 100 °C, the elastic modulus of GFRP bars was reduced by 1.5%, 1.1% and 1.3% at 0 h, 1 h and 2 h, respectively. At 200 °C, the elastic modulus of GFRP bars was reduced by 4.7%, 1.8% and 16.3% at 0 h, 1 h and 2 h, respectively. Further, at 300 °C, the elastic modulus of GFRP bars was reduced by 6.0%, 9.8% and 18.3% at 0 h, 1 h and 2 h, respectively. These results showed that the higher the exposure temperature was, the more degradation effect of holding time on GFRP bars was. On the whole, within the experimental temperature range, the elastic modulus of GFRP bars decreased within 19%, while the tensile strength of GFRP bars was reduced by 28.0%. The drop of elastic modulus of GFRP bars was lower than that of the tensile strength, which indicated that the degradation of the elastic modulus of GFRP after elevated temperature exposure was lower than that of tensile strength. This phenomenon may be related to the deterioration mechanism of GFRP bars after elevated temperatures exposure. The main factor influencing the elastic modulus of FRP bars was the modulus of fibers in the FRP bars [41]. Since the elevated temperatures in this study may not reach the softening temperature of glass fibers, the degradation only occurred for the resin and the interface adhesion between the resin and the fibers in GFRP bars. Therefore, the decrease of elastic modulus of GFRP bars was relatively not so high. However, with the resin vitrified or carbonized, the tensile strength of GFRP bars would decrease obviously. These test results are similar to those reported in References [19,20,22].

### 4.2. Tensile Fatigue Properties of GFRP Bars after Elevated Temperatures Exposure

#### 4.2.1. Influence of Cyclic Load on the Tensile Strength of GFRP Bars

**Figure 6:**
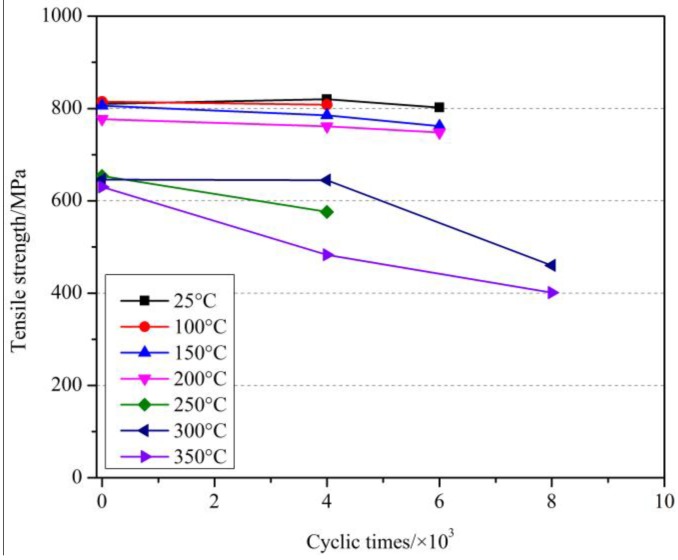
The effect of cyclic load on the tensile strength of GFRP bars after elevated temperatures with a holding time of 0 h.

**Figure 7:**
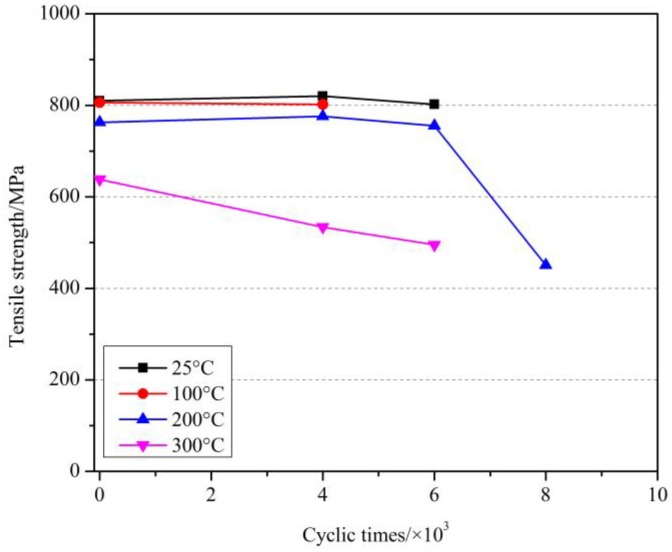
The effect of cyclic load on the tensile strength of GFRP bars after elevated temperatures with a holding time of 1 h.

**Figure 8:**
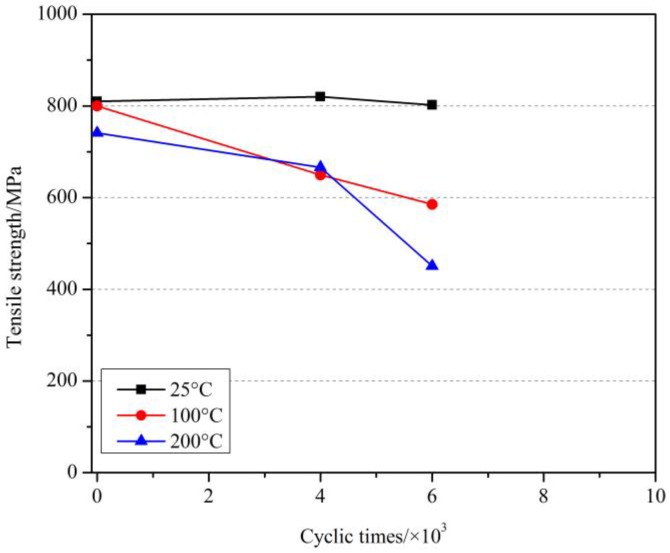
The effect of cyclic load on the tensile strength of GFRP bars after elevated temperatures with a holding time of 2 h.

Since the fatigue life of GFRP bars at room temperatures was much less than 2 million under the given test conditions, we mainly focused on the effects of cyclic loading on the tensile strength and elastic modulus in this study. Table 3 shows the tensile test results of GFRP bars under the cyclic load after elevated temperatures exposure. Figure 6, Figure 7 and Figure 8 showed the effect of cyclic load on the tensile strength of GFRP bars after elevated temperatures with holding time of 0 h, 1 h and 2 h.

As can be seen from Figure 6 and Table 3, at room temperature, when the number of cyclic load was 6000, the tensile strength of GFRP bars decreased only by 1.0%, which revealed that the effect of cyclic load was slight. At 150 °C, compared with the specimens without cyclic load at 150 °C and at room temperature, the tensile strength of GFRP bars after 4000 cyclic loads decreased by 2.6% and 3.1%, respectively, while after 6000 cyclic loads decreased by 5.5% and 5.9%, respectively. Further, at 350 °C, compared with the specimens without cyclic load at 350 °C and at room temperature, the tensile strength of GFRP bars after 4000 cyclic loads decreased by 23.3% and 40.4%, respectively, while after 8000 decreased by 36.5% and 50.5%, respectively. It can be concluded that the cyclic load had a deteriorating effect on the GFRP bars after elevated temperatures exposure, and the tensile strength of GFRP bars decreased more with the increased number of the cyclic load. At the same time, it can be obtained that the deterioration degree of the cyclic load on the GFRP bars was enhanced with the increase of the elevated temperature. That is, the higher the elevated temperature was, the more serious the damage of GFRP bars suffered from elevated temperature was, and the more obvious the deterioration of the tensile strength of GFRP bars caused by the cyclic load was.

The effect of cyclic load on the tensile strength of GFRP bars after elevated temperatures with a holding time of 1 h are shown in Figure 7. Similarly, at 200 °C, compared with the specimens without cyclic load at 200 °C and at room temperature, the tensile strength of GFRP bars after 6000 cyclic loads decreased by 1.0% and 6.8%, respectively, while after 8000 cyclic loads decreased by 40.9% and 44.3%, respectively. At 300 °C, compared with the specimens without cyclic load at 300 °C and at room temperature, the tensile strength of GFRP bars after 4000 cyclic loads by 16.3% and 34.1%, respectively, while after 6000 cyclic loads decreased by 22.4% and 38.9%, respectively. Further, the comparison results for the holding time of 2 h are shown in Figure 8. More comparison for the effect of holding time will be discussed later.

**Figure 9:**
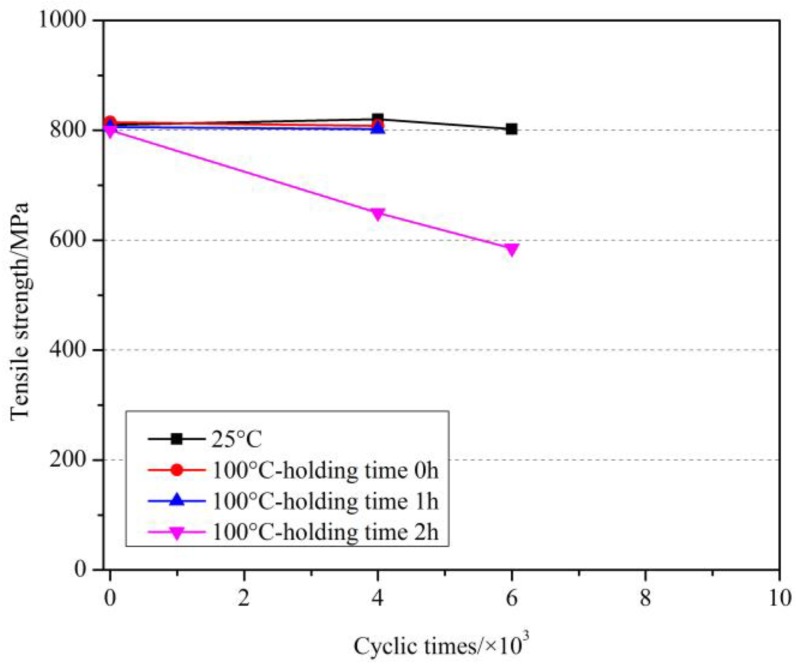
The effect of cyclic load on the tensile strength of GFRP bars at 100 °C.

Figure 9 shows the effect of cyclic load on the tensile strength of GFRP bars at 100 °C with different holding times. As can be seen from Figure 9 and Table 3, at 100 °C, compared with the specimens without cyclic load at 100 °C (or at room temperature), the tensile strength of GFRP bars after 4000 cycles of loading decreased by 0.9%, 0.5% and 18.8% (or 0.2%, 1% and 19.8%) with a holding time of 0 h, 1 h and 2 h respectively. It was clear that the holding time of 0 h and 1 h at 100 °C slightly decreased the tensile strength of GFRP bars, whereas the holding time of 2 h obviously decreased the tensile strength. Meanwhile, after 8000 cycles of loading, the tensile strength reduced by 27% (or 28%) than without cyclic load at 100 °C (or at room temperature). As can be obtained that with the holding time increased, the degradation effect of cyclic load on GFRP bars increased gradually.

**Figure 10:**
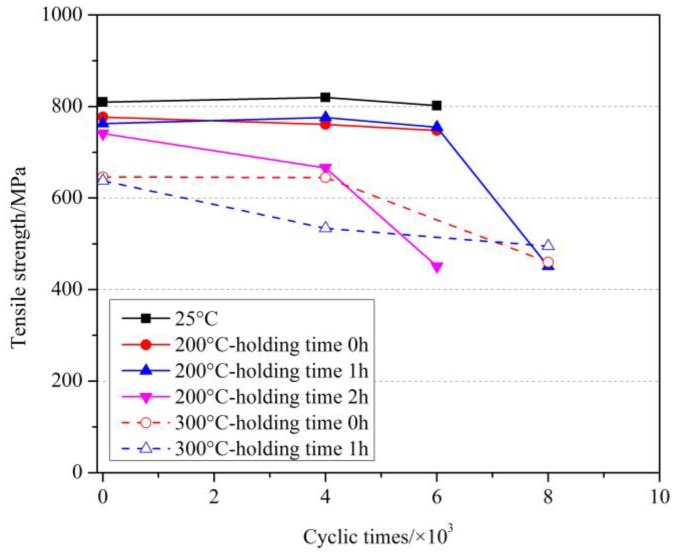
The effect of cyclic load on the tensile strength of GFRP bars at 200 and 300 °C.

Figure 10 further compared the results of 200 °C and 300 °C with different holding times. At 200 °C, compared with the specimens without cyclic load at 200 °C (or at room temperature), the tensile strength of GFRP bars after 4000 cycles of loading decreased by 2.1%, −1.7% and 10.1% (or 6.0%, 4.2% and 17.8%) with a holding time of 0 h, 1 h and 2 h respectively, and after 6000 cycles of loading decreased by 3.7%, 1.0% and 39.1% (or 7.7%, 6.8% and 44.3%) with a holding time of 0 h, 1 h and 2 h respectively. It is noted that the odd value (i.e., −1.7%) may be caused by the coefficient of variation of test results. Similarly, at 300 °C, compared with the specimens without cyclic load at 200 °C (or at room temperature), the tensile strength after 4000 cycles of loading reduced by 0.2% and 16.3% (or 20.4% and 34.1%) with a holding time of 0 h and 1 h respectively, and after 8000 cycles of loading reduced by 28.8% and 22.4% (or 43.2% and 38.9%) with a holding time of 0 h and 1 h respectively. 

Meanwhile, the results in Figure 9 and Figure 10 also show the combination of different holding times and elevated temperatures could lead to the same reduction of tensile strength of GFRP bars (e.g., after 4000 cycles in 200 °C–2 h and 300 °C–0 h cases). This proved that the coupling of holding time and elevated temperature could enhance the degradation effect of cyclic load on the tensile strength of GFRP bars.

In brief, the cyclic load had a strong deterioration effect on the GFRP bars after the elevated temperature exposure, especially when the exposure temperature was more than 200 °C. The possible explanation for this phenomenon was as followings. GFRP bars were composed of resin and glass fibers. When the temperature and holding time were both relatively low (e.g., 100 °C–0 h and 100 °C–1 h), the glass transition state of resin could not be caused by the elevated temperature. In this case, the resin was still in vitreous state and its property did not decrease sharply. Therefore, the tensile strength of GFRP bars after cyclic load did not change significantly. When the elevated temperature could cause carbonization of the resin (e.g., ≥250 °C), even if the specimen returned to room temperature after the elevated temperature treatment, the mechanical properties of resin and its bond to glass fibers could not be fully recovered, resulting in the reduction of the resistance of glass fiber to the tensile load. Under cyclic load, the dispersed glass fibers were not evenly distributed and more easily broken, thus weakening the effective tensile area of GFRP bars, and this situation occurred continuously under cyclic load until the GFRP bars were broken and damaged. In this case, the elevated temperature may also reduce the tensile strength of glass fiber, so the tensile strength of GFRP bars after elevated temperature exposure under cyclic load was sharply reduced. In the other hand, with the extension of holding time (from 0 h to 1 h, and then to 2 h) at given elevated temperature, the defects inside GFRP bars (such as micro-cracks, void caused by degradation of resin and interface area) should increase gradually. Especially when the holding time reached 2 h, the content of defects may hugely increase due to the intrinsic nature of the resin at elevated temperature environment. Therefore, the cyclic load finally accelerated the development of defects, resulting in the lower tensile strength of GFRP bars.

#### 4.2.2. Influence of Cyclic Load on the Elastic Modulus of GFRP Bars

**Figure 11:**
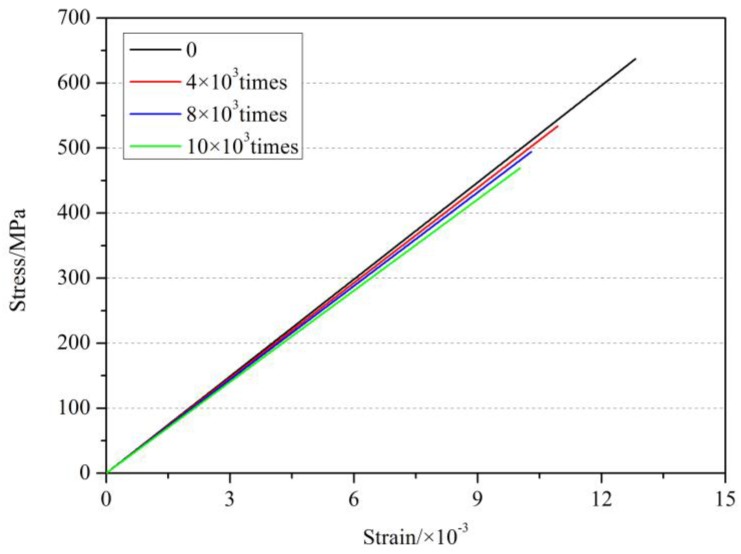
Stress-strain curve of GFRP bars under cyclic load at 350 °C and holding time of 0 h.

Figure 11 shows the stress-strain curves of GFRP bars under cyclic load at 350 °C and holding time of 0 h. As found in Figure 11 and Table 3, the tensile elastic modulus of GFRP bars after elevated temperatures decreased after the different times of cyclic load. When the number of fatigue loading cycles was low, the decrease of the tensile elastic modulus of GFRP bars was not obvious. With the increase of the number of fatigue loading cycles, the tensile elastic modulus of GFRP bars tended to reduce after elevated temperature exposure. The tensile elastic modulus of GFRP bars at 200 °C and holding time of 1 h after 14,000 cycles of loading decreased by 10.9% than that at room temperature and by 9.2% than that after exposing at 200 °C and holding time of 1 h without cyclic load. The tensile elastic modulus of the GFRP bars at 350 °C after the cyclic load decreased by 17.6% than that at room temperature and by 6.0% than that after exposing at 350 °C without cyclic load. The reasons for these phenomenon of reduction of elastic modulus of GFRP bars may be that the matrix resin of FRP bars was vitrified or carbonized after the elevated temperature exposure (e.g., ≥200 °C), and then the properties and bonding ability of the resin were reduced. Then the ability of GFRP bars to resist the fatigue loading was weakened, which finally lead to the decrease of the elastic modulus of the GFRP bars under cyclic load.

## 5. Conclusions

A total of 105 GFRP bars tensile specimens were constructed and tested to failure. The test parameters included the elevated temperature (25, 100, 150, 200, 250, 300 and 350 °C), holding time (0, 1 and 2 h), and the number of cyclic load (4000, 6000, 8000, 10,000 and 14,000). Based on the experimental results, the following conclusions can be drawn:

(1) With the increase of elevated temperature and holding time, the tensile strength and elastic modulus of GFRP bars both tended to decrease. When the temperature exceeded 200 °C, the tensile strength of GFRP bars decreased dramatically and the deterioration effect of holding time was significant.

(2) The deterioration effect of elevated temperature on the tensile strength of GFRP bars was obviously higher than that on the elastic modulus. Within the test temperature range (i.e., 25–350 °C), the tensile strength and elastic modulus of GFRP bars were reduced by 28.0% and 18.3%, respectively.

(3) The cyclic load accelerated the degradation of GFRP bars after elevated temperature exposure. The defects caused by the elevated temperature treatment were aggravated by the cyclic load, finally resulting in the lower tensile strength of GFRP bars.

(4) The coupling of elevated temperature and holding time enhanced the degradation effect of cyclic load on GFRP bars. With the increase of the temperature and holding time, GFRP bars suffered more severe thermal damage, and the degradation effect of the cyclic load on the tensile strength of GFRP bars was more obvious.

(5) The deterioration effect of cyclic load on the elastic modulus of GFRP bars after elevated temperature exposure was obvious, and the tensile elastic modulus reduced by a maximum of 17.6% compared with that without cyclic load at room temperature.

## Figures and Tables

**Table 1:** Mechanical properties of glass fiber-reinforced polymer (GFRP) bars.

Type of FRP Bars	Diameter/mm	Ultimate Tensile Strength/MPa	Elastic Modulus/GPa
GFRP	12	810	55.1

**Table 2:** Designation of tested specimens.

**Temperatures/°C**	25	100	150	200	250	300	350
**Holding Time/h**	-	0, 1, 2	0	0, 1, 2	0, 1, 2	0, 1, 2	0

**Table 3:** The tensile results of GFRP bars after cyclic load.

Temperature/°C	Holding Time/h	Cyclic Times	Tensile Strength/MPa	Elastic Modulus/GPa
25	/	0	810	55.1
		4000	820	55.2
		6000	802	54.5
100	0	0	815	54.3
		4000	808	54.6
100	1	0	806	54.5
		4000	802	53.9
100	2	0	800	54.4
		4000	650	52.6
		8000	585	54.7
150	0	0	806	53.2
		4000	785	53.8
		6000	762	53.4
200	0	0	777	52.5
		4000	761	53.6
		6000	748	53.6
200	1	0	763	54.1
		4000	776	55.6
		6000	755	53.1
		8000	451	54.6
		14,000	439	49.1
200	2	0	741	46.4
		4000	666	45.7
		6000	451	45.2
250	0	0	652	53.9
		4000	576	53.6
300	0	0	646	51.8
		4000	645	50.9
		8000	460	50.7
300	1	0	638	49.7
		4000	534	48.8
		8000	495	48.0
		10,000	469	46.8
350	0	0	630	48.3
		4000	483	47.2
		8000	401	45.4
